# Devenir sérologique et nutritionnel des nourrissons nés de mères séropositives suivis dans l’option B+ à Guédiawaye

**DOI:** 10.11604/pamj.2016.25.224.10441

**Published:** 2016-12-07

**Authors:** Diouf Jean Baptiste, Diallo Djibril, Sylla Assane, Mbaye Ngagne, Ouattara Baly, Ndiaye Ousmane

**Affiliations:** 1 Centre Hospitalier Roi Baudouin de Guédiawaye, Dakar, Sénégal; 2 Clinique Gynécologique et Obstétricale, Hôpital Aristide Le Dantec Dakar. Avenue Pasteur, BP: 3001 Dakar, Sénégal; 3 Université Cheikh Anta Diop de Dakar Sénégal; 4 ONG Synergie pour l’enfance, Cité Lobatt Fall Pikine, BP: 20330 Thiaroye

**Keywords:** PTME, VIH, option B+, nutrition, PMTCT, HIV, option B +, nutrition

## Abstract

Dans le cadre de son plan d’élimination de la transmission de la mère à l’enfant du VIH, le Sénégal a adopté depuis 2012 l’option B+ de l’OMS consistant en une mise sous trithérapie systématique des femmes enceintes séropositives, associée à l’allaitement maternel et la mise sous prophylaxie antirétrovirale (ARV) de leurs nourrissons. L’objectif de notre étude était d’étudier les risques de transmission mère - enfant du VIH et le devenir nutritionnel des nourrissons dans l’option B+. Il s’agissait d’une étude rétrospective descriptive réalisée du 1^er^ septembre 2012 au 30 avril 2015 au Centre Hospitalier Roi Baudouin de Guédiawaye. Ont été inclus tous les nourrissons dont les mères ont été sous trithérapie, ayant bénéficié d’un allaitement maternel protégé et d’une prophylaxie ARV et chez qui la sérologie du 14^ème^ mois a été réalisée. Les paramètres étudiés étaient l’âge et le profil sérologique de la mère, le statut sérologique du père, le partage du statut au sein du couple, la conduite de l’alimentation du nourrisson, la prophylaxie ARV reçue par le nourrisson, l’état nutritionnel à 6 et 12 mois et le statut sérologique du nourrisson à 14 mois. Sur 126 nourrissons suivis dans le cadre de la PTME, 42 soit 33,33% respectant les directives de l’option B+ ont été inclus dans l’étude. L’âge des mères variait entre 15 et 42 ans avec une moyenne de 31 ans. La majorité des mères (88,1%) étaient porteuses du virus de type 1 et 11,9% de type 2; 20 couples (47,62%) étaient séroconcordants, 14 sérodifférents tandis que le statut sérologique était inconnu ou non investigué chez 8 pères (19,05%). Une différence significative entre le profil sérologique des pères et le partage (p<0,05) était retrouvée. La majorité des enfants (88,1 %) était née par voie basse à terme (95,2%), avec un poids de naissance moyen de 2880 grammes. Concernant la prophylaxie reçue, la majorité des nourrissons était sous monothérapie prophylactique, 27(64,28%) sous NVP, et 4 (9,52%) sous AZT, alors que 11 (26,19%) étaient sous trithérapie à base de AZT+3TC+NVP. L’AME était effectif chez 80,9% des nourrissons et le sevrage à 12 mois chez 80,9% des nourrissons. Sur le plan nutritionnel, à 6 mois 12% et 7,1% avaient respectivement une MAM et une MAS. A 12 mois 19,1% avaient une MAM. La sérologie rétrovirale était négative chez l’ensemble des 42 nourrissons à 14 mois. L’option B+ reste une stratégie de lutte efficace pour réduire le taux de la TME. Cependant la malnutrition précoce des nourrissons exige un soutien nutritionnel des mères allaitantes de même qu’un accompagnement psychosocial de qualité.

## Abstract (en)

As part of its Plan to eliminate mother-to-child transmission of HIV, Senegal has adopted, since 2012, WHO's B + option, which consists of systematic triple therapy for HIV-positive pregnant women associated with breastfeeding and antiretroviral (ARV) prophylaxis for their infants. Our study aims to analyze the risks of mother-to-child transmission of HIV and the nutritional outcome of infants undergoing B + option. Material and methods: We conducted a descriptive, retrospective study at the King Baudouin health center in Guédiaway from 1 September 2012 to 30 April 2015. All infants whose mothers were on triple therapy, undergoing protected breastfeeding, ARV prophylaxis and serological test at 14th months were included in the study. The parameters studied were mother's age and serological profile, father's serological status, the sharing of the status within the couple, infant nourishing, infant ARV prophylaxis, nutritional status at 6 and 12 months and serological status of the infant at 14 months. Out of the 126 infants undergoing PMTCT program, 42 or 33.33% of infants following the B + guidelines were included in the study. The age of mothers ranged from 15 to 42 years, with an average age of 31 years. The majority of mothers (88.1%) carried type 1 virus and 11.9% carried type 2 virus; 20 couples (47.62%) were sero-concordant, 14 were serodifferent, while the serological status was unknown or not investigated in 8 fathers (19.05%). A significant difference between fathers' serological profile and the sharing status (p <0.05) was found. The majority of infants (88.1%) were born at term via vaginal delivery (95.2%), with an average birth weight of 2880 grams. In relation to prophylaxis, the majority of infants received prophylactic monotherapy, 27 (64.28%) received NVP, 4 (9.52%) received AZT, while 11 (26.19%) received triple combination of AZT + 3TC + NVP. The AME was effective in 80.9% of infants and weaning began at 12 months in 80.9% of infants. In relation to nutritional status, at 6 months 12% and 7.1% of infants had MAM and MAS respectively. At 12 months, 19.1% of infants had MAM. Retroviral serology was negative in all the 42 infants at 14 months. B + option is an effective strategy to reduce the MTCT rate. However, early malnutrition in children requires nutritional support for breastfeeding mothers as well as a good psychosocial support.

## Introduction

La transmission du VIH de la mère à l'enfant est la principale voie de contamination pédiatrique. Cette transmission peut survenir in utero dans les dernières semaines de la grossesse, au moment de l'accouchement et/ou durant l'allaitement. La prévention de la transmission mère-enfant (PTME) du VIH est un domaine dynamique qui évolue rapidement. Les dernières mises à jour de l'OMS dans le cadre de la PTME remontent au mois d'avril 2012 [1]. L'OMS recommande aux pays à ressources faibles ou intermédiaires d'instaurer une trithérapie à vie chez les femmes enceintes séropositives [1]. Au Sénégal, la PTME a connu une évolution dynamique depuis la phase pilote entre 2000 et 2002 au niveau de trois sites (Centre de santé Roi Baudouin, Hôpital Aristide Le Dantec et Hôpital Principal de Dakar) jusqu´à l'adoption en 2012 de l'option B+ pour l'atteinte des objectifs d'élimination de la transmission mère-enfant du VIH à l'horizon 2015. Cette option consiste à une mise sous trithérapie à vie de la femme enceinte séropositive dépistée dans le cadre de la consultation prénatale, mise sous prophylaxie antirétrovirale de l'enfant né de mère séropositive et un allaitement maternel protégé. Il demeurait des interrogations et des appréhensions quant au risque résiduel de TME via le lait maternel, à l'observance du traitement antirétroviral, à l'état nutritionnel des mères allaitantes et de leurs enfants et à la charge de travail des prestataires. C'est dans cette optique que nous avons mené cette étude qui avait pour objectifs d'évaluer les risques de transmission de la mère à l'enfant du VIH et le devenir nutritionnel des enfants dans l'option B+.

## Méthodes

Nous avons réalisé une étude rétrospective descriptive du 1^er^ /09/2012 au 30/04/2015 au Centre Hospitalier Roi Baudouin. Ce centre hospitalier est un établissement public de santé de niveau 1 situé dans la banlieue de Dakar et disposant d'un service de pédiatrie. La prise en charge des enfants vivant avec le VIH s'y effectue en collaboration avec l'ONG Synergie pour l'enfance qui est une Organisation Communautaire de Base née en 1996, engagée dans la prévention de la transmission mère-enfant et dans la prise en charge des enfants affectés par le VIH. Nous avons inclus dans cette étude les nourrissons qui ont bénéficié de l'option B+ et chez qui la sérologie du 14^ème^ mois était disponible.

Selon l'option B+, les femmes enceintes dépistées séropositives au cours de la consultation prénatale étaient systématiquement mises sous traitement antirétroviral à vie. Les nouveaux nés de mères séropositives allaitantes étaient sous trithérapie. La chimioprophylaxie au cotrimoxazole était effective à partir de 6 semaines et l'allaitement maternel arrêté à 12 mois. Des visites régulières étaient réalisées avec un bilan clinique et biologique et une sérologie proposée à 14 mois. Nous avons étudié les paramètres suivants: l'âge de la mère réparti en différentes tranches: < 20 ans, [20-24], [25-29], [35-40], et > 40 ans; le profil sérologique de la mère : VIH1, VIH2 ou VIH1 et VIH2; le statut sérologique du père : positif (couple séroconcordant), négatif (couple sérodifférent), inconnu ou alors non testé; le partage et connaissance du statut sérologique au VIH du partenaire : oui ou non; l'âge gestationnel : A terme entre [37 et 42 SA], prématuré avant 37SA, post mature après 42 SA; la voie d'accouchement : basse ou césarienne; la prophylaxie à la naissance : Névirapine (NVR), Zidovudine (AZT) ou trithérapie [AZT+3TC(Lamivudine) +NVP ou AZT+3TC + LVP/r (Lopinavir/ritonavir)]; le poids à la naissance réparti en différentes tranches : <2500g, [2500g-3000g], [3000g-3500g], >3500g; la diversification de l'alimentation à 6 mois; le sevrage à 12 mois; l'état nutritionnel à 6 mois évalué par l'indice poids / taille; l'état nutritionnel à 12 mois évalué par l'indice poids/taille; le profil sérologique des nourrissons à 14 mois.

Les données étaient analysées à l'aide du logiciel épi info 2000. Nous avons présenté les variables qualitatives sous forme de proportions avec leur intervalle de confiance à 95%. Les quantitatives étaient présentées sous forme de moyenne.

## Résultats

Nous avons inclus dans cette étude 42 nourrissons nés de mères séropositives, qui respectaient les directives de l'option B+ et avec une sérologie du 14^ème^ mois disponible sur 126 nourrissons suivis dans le cadre de la PTME soit 33,33% des cas.

### Caractéristiques des gestantes séropositives et de leurs conjoints (Tableau 1)

L'âge moyen des mères était de 31,45 ans avec des extrêmes allant de 15 et 42 ans. La tranche d'âge la plus représentée était entre [25-29] ans. La majorité des mères 37 soit 88,10% étaient infectées par le VIH1, et 5 mères étaient infectées par le VIH2 soit 11,90%. Il n'y avait pas de co-infection VIH1/VIH2 (Tableau 1). Dans notre série 20 couples (47,62%) étaient séroconcordants, 14 séronégatifs tandis que le statut sérologique était inconnu ou non investigué chez 8 (19,05%) pères. Le partage du statut au sein du couple était effectif dans 71,43% des cas.

**Tableau 1:** Caractéristiques des parents

variables	effectif	pourcentage	IC à 95 % des pourcentages
**Tranche d’âge**			
<20 ans	1	2,38	0,06 - 12,57
20 ans - 24 ans	2	4,76	0,58 - 16,6
25 ans - 29 ans	16	38,10	23,57 - 54,36
30 ans - 34 ans	10	23,81	12,05 - 39,45
35 ans - 40 ans	7	16,67	6,97 - 31,36
> 40 ans	6	14,29	5,43 - 28,54
**Profil sérologique des mères**			
VIH 1	37	88,10	74,37 - 69,02
VIH 2	5	11,90	3,98 - 25,63
**Profil sérologique des pères**			
séropositif	14	33,33	19,57 - 49,55
Séronégatif	20	47,62	32,00 - 63,58
Inconnu /non testé	8	19,05	8,60 - 34,12
**Partage du statut**			
Non	12	28,57	15,72 - 44,58
oui	30	71,43	55,42 - 84,28

### Caractéristiques et données concernant les nouveaux nés (Tableau 2)

La majorité des nouveaux nés 37 (88,10%) était née par voie basse et à terme 40(95,24%). Le poids moyen à la naissance était de 2290g avec des extrêmes allant de 2000g à 3700g. Les nourrissons dont le poids était compris entre 3000g et 3500g et entre 2500g et 3000g étaient les plus représentatifs avec respectivement 35,71% et 33,33%. La majorité des nouveaux nés étaient sous monothérapie prophylactique, 27 cas (64,28%) sous NVP, et 4 (9,52%) sous AZT, alors que 11 (26,19%) étaient sous trithérapie à base de AZT+3TC+NVP. Tous les nourrissons de notre série étaient sous allaitement maternel exclusif, la diversification à 6 mois était effective chez 34 nourrissons (88,95%) et le sevrage à 12 mois chez 34 nourrissons (88,95%). Dans notre série, 5 nourrissons (11,90%) avaient une malnutrition aigüe modérée et 3 (7,14%) avait une malnutrition aiguë sévère à 6 mois. A 12 mois la majorité des nourrissons avaient un état nutritionnel normal et 8 (19,05%) une malnutrition aigüe modérée. A 14 mois, la sérologie était négative chez l'ensemble des 42 nourrissons, soit un taux de transmission mère enfant nul (Tableau 2).

**Tableau 2:** Caractéristiques des nourrissons

variables	effectif	pourcentage	IC à 95 % des pourcentages
Voie d’accouchement			
Basse	37	88,10	74,37 - 96,02
césarienne	12	11,90	3,98 - 25,63
Maturité			
Prématuré	2	4,76	0,58 - 16,16
A terme	40	95,24	83,84 - 99,42
Poids de naissance			
< 2500 g	9	21,43	10,30 - 36,18
2500 g - 3000 g	14	33,33	19,57 - 49,55
3000 g - 3500 g	15	35,71	21,55 - 51,97
> 3500 g	4	9,52	2,66 - 22,62
Prophylaxie reçue à la naissance			
NVP	27	64,28	49,36 - 79,28
AZT	4	9,52	2,66 - 22,62
AZT+3TC+NVP	11	26,19	11,19 - 41,27
Diversification à 6 mois			
Non	8	19,05	8,60 - 34,12
Oui	34	80,95	65,88 - 91,40
Sevrage à 12 mois			
Non	8	19,05	8,60 - 34,12
Oui	34	80,95	65,88 - 34,12
Etat nutritionnel à 6 mois			
Normal	34	80,95	65,88 - 91,40
MAM	5	11,9	3,98 - 25,63
MAS	3	7,14	1,50 - 19,48
Etat nutritionnel à 12 mois			
Normal	34	80,95	65,88 - 91,40
MAM	8	19,05	8,60 - 34,12
MAS	0	0	0,00 - 0,00

**MAS** = malnutrition aigüe sévère

## Discussion

Notre étude s'est déroulée sur une période de 32 mois et avait pour objectif d'évaluer le devenir sérologique et nutritionnel des nourrissons nés de mères séropositives dans l'option B+. Sur l'ensemble des 126 nourrissons suivis durant cette période dans le cadre de la PTME seuls 42 nourrissons respectaient les directives de l'option B+ avec une sérologie du 14 ème mois disponible soit un taux de 33,33%. Ce faible taux d'inclusion peut être expliqué d'une part par la réticence des mères par rapport au risque résiduel de transmission via le lait maternel et d'autre part par la disponibilité gratuite des substituts de lait à travers des programmes et organisations non gouvernementales (ONG) dans la localité.

### Caractéristiques des gestantes séropositives et de leurs conjoints

Les mères étaient relativement jeunes. La tranche d'âge entre [25-29] ans était plus représentative avec 54, 36%. Ces résultats sont superposables à ceux retrouvés dans des travaux au Sénégal [2]. Par contre dans une étude faite au Bénin portant sur VIH et PTME par l'option B de l'OMS, la tranche d'âge la plus représentée était de [20-35] ans [3]. Cet âge moyen relativement jeune dans notre série pourrait s'expliquer par l'importance de l'exposition des femmes en âge de procréer à la transmission hétérosexuelle qui est le principal mode de transmission dans nos régions. Concernant le profil sérologique des mères, nous avons noté une prédominance du VIH 1 avec 88,10% et aucun cas de co-infection n'a été retrouvé. La plus grande transmission du VIH1 pourrait expliquer la prédominance de ce sérotype. Nous avons noté un taux élevé d'accouchements par voie basse 37 cas (88,10%). Une étude antérieure menée dans la même structure [2] a abouti à des résultats similaires, avec 95% d'accouchement par voie basse, et le lien entre l'effet protecteur de la césarienne, et le statut sérologique définitif des enfants n'avait pas pu être affirmé par cette étude. Ces résultats sont également identiques à ceux obtenus dans d'autres travaux au Sénégal [4] et au Togo [5]. Aucune influence du mode d'accouchement sur le risque de transmission n'avait été retrouvée. Dans notre série toutes les mères étaient sous trithérapie durant la grossesse conformément aux directives de l'option B+. Des études faites en France et en Thaïlande ont prouvé l'efficacité du traitement ARV au cours de la grossesse avec un taux de transmission qui est passé de 15 à 1% au bout de 10 ans en France [6]. L'administration des ARV au cours de la grossesse et de l'accouchement réduit efficacement la transmission du virus de la mère à l'enfant [7]. Ces traitements réduisent le risque de la TME en diminuant la réplication du virus chez la mère d'une part, et d'autre part en assurant une prophylaxie chez le nourrisson pendant et après son exposition au virus [7]. Dans notre série, 20 couples (47,62%) étaient séroconcordants, 14 sérodifférents, tandis que le statut sérologique était inconnu ou non testé chez 8 pères (19,05%). Au Togo parmi 322 pères inclus dans une cohorte, seuls 156 cas connaissaient leur statut sérologique dont 139 étaient séropositifs soit 89,1% [5]. On peut expliquer les cas non testés ou inconnus par l'inégalité de l'implication dans le programme de PTME entre homme et femme [8].

### Caractéristiques et données concernant les nouveaux nés

La majorité des nouveaux nés étaient nés à terme dans 40 cas (95,24%), et concernant le poids la tranche comprise entre [2500-3500] grammes était la plus représentative (69,04%). Les faibles poids représentaient 21,43% dans notre série. Ces résultats sont comparables à ceux retrouvés au Sénégal [2, 9], et au Togo [5]. Dans notre série, la majorité des nourrissons étaient sous monothérapie prophylactique, 27 cas (64,28%) sous NVP et 4 cas (9,52%) sous AZT. Le reste des nourrissons (26,19%) était sous trithérapie à base de AZT+3TC+NVP. Les recommandations nationales préconisent une trithérapie chez les nourrissons. Notre étude s'est déroulée durant la phase de transition du passage de l'option B à l'option B+ marquée par la non disponibilité des doses combinées d'ARV pédiatrique. Ceci justifie qu'une proportion de nourrisson ait été mise sous monothérapie. Ces résultats sont encourageants, car ils montrent la possibilité d'une prévention post-natale de la contamination avec les antirétroviraux qui respecte la tradition de l'allaitement au sein. Tous les 42 nourrissons sont séronégatifs 2 mois après le sevrage complet soit un taux de transmission nul. Une étude faite au Bénin portant sur l'évaluation de l'option B avait montré un taux de transmission de 2,9% [3]. De même au Togo dans une étude où 96,3% des mères étaient sous prophylaxie et 99,6% des nouveaux nés sous ARV, le taux de transmission était de 9% avec 4,58% chez les nourrissons qui étaient sous allaitement maternel exclusif et 5,85% pour ceux qui étaient sous allaitement artificiel [5]. En chine dans une méta-analyse où moins de 1% des nourrissons avaient reçu une trithérapie, le taux de transmission mère enfant du VIH était de 4,4%. [10]. Ces différents résultats confirment l'efficacité de l'option B+ dans la prévention de la transmission mère enfant du VIH.

En ce qui concerne l'alimentation, tous les nourrissons de notre série étaient sous allaitement maternel exclusif, la diversification à 6 mois était effective chez 34 nourrissons (88,95%) et le sevrage à 12 mois chez 34 nourrissons (88,95%). Ces chiffres s'opposent à d'autres retrouvés au Sénégal [9, 11], où la majorité des enfants étaient nourris par un substitut de lait maternel. Ceci s'explique aisément par le changement de politique d'alimentation dans l'option B+ où l'allaitement maternel protégé est fortement indiqué. Ils sont supérieurs à ceux retrouvés au Togo (39,08%.) [5] et au Bénin (59,9%) [3]. Par contre l'allaitement au sein reste déconseillé chez la femme vivant avec le VIH en France comme dans les pays industrialisés. [12]. Dans la plupart des pays subsahariens, l'OMS recommandait l'allaitement maternel, car le risque de décès de l'enfant par malnutrition et par des maladies infectieuses létales autres que le VIH serait supérieur au risque infectieux du VIH, du coût encore élevé du lait de substitution et des conditions d'hygiène devant encadrer un allaitement artificiel [13, 14]. Par ailleurs l'exclusivité de l'allaitement maternel jusqu'à 6 mois dans notre étude (88,95%) est supérieur à la moyenne nationale révélé dans l'enquête démographique et de santé (EDS) continue 2012-2013 (38%) [15]. Les obstacles au sevrage à 12 mois peuvent s'expliquer par le faible pouvoir décisionnel des mères et les craintes d'une stigmatisation. Au Sénégal selon l'EDS continue 20112-2013, 97 % d'enfants sont encore au sein à 12-15 mois.

Dans notre série 11,90% des nourrissons avaient une malnutrition aigüe modérée et 7,14% une malnutrition aigüe sévère à 6 mois. A 12 mois la majorité des nourrissons avaient un état nutritionnel normal et 19,05% avaient une malnutrition aigüe modérée. La survenue précoce de malnutrition aigüe sévère à 6 mois et le taux élevé de malnutrition aigüe modérée à 12 mois comparés aux résultats de l'EDS continue 2012-2014 posent le problème de l'état nutritionnel des mères allaitantes. La malnutrition pourrait débuter depuis la vie intra-utérine, du fait que les personnes vivant avec le VIH ont des troubles nutritionnels fréquents. Ces troubles nutritionnels affecteraient probablement le poids de l'enfant. La faible cohorte pourrait constituer une limite à notre étude. Une extension du nombre de cas pourrait permettre de mieux apprécier l'option B+ et les problèmes nutritionnels.

## Conclusion

Le bilan des activités de PTME au centre hospitalier Roi Baudouin de Guédiawaye s'est révélé concluant. Le taux de transmission mère enfant du VIH est nul. Ce qui prouve l'efficacité de l'Option B+ dans le cadre de la PTME. Cependant la malnutrition précoce des nourrissons exige un soutien nutritionnel des mères allaitantes de même qu'un accompagnement psychosocial de qualité.

### Etat des connaissances actuelle sur le sujet

- La transmission verticale est le principal mode de transmission du VIH chez l'enfant. Interrompre cette transmission peut contribuer à atteindre les objectifs d'élimination de l'épidémie du VIH d'ici l'horizon 2030;
- Dans le cadre de son plan d'élimination de la transmission de la mère à l'enfant du VIH, le Sénégal a adopté depuis 2012 l'option B+ de l'OMS consistant en une mise sous trithérapie systématique des femmes enceintes séropositives, associée à l'allaitement maternel et la mise sous prophylaxie antirétrovirale (ARV) de leurs nourrissons;
- La malnutrition est un facteur de comorbidité du VIH.

### Contribution de notre étude à la connaissance

- l'option B+ reste une stratégie efficace et reproductible dans les PVD pour la prévention de la transmission mère - enfant du VIH;
- La survenue de la malnutrition précoce chez les nourrissons suivis dans notre cohorte exige un soutien nutritionnel des mères allaitantes et un accompagnement psychosocial de qualité;
- Faire la promotion de l'allaitement maternel protégé et l'implication des pères dans les programmes de PTME.
